# Cross-modal auditory priors drive the perception of bistable visual stimuli with reliable differences between individuals

**DOI:** 10.1038/s41598-021-96198-7

**Published:** 2021-08-20

**Authors:** Zsófia Pálffy, Kinga Farkas, Gábor Csukly, Szabolcs Kéri, Bertalan Polner

**Affiliations:** 1 Department of Cognitive Science, Budapest University of Technology and Economics, 1 Egry József utca, Building T, Floor 5, Budapest, 1111 Hungary; 2 Department of Psychiatry and Psychotherapy, Semmelweis University, Budapest, Hungary; 3 National Institute of Psychiatry and Addictions, Budapest, Hungary

**Keywords:** Perception, Human behaviour

## Abstract

It is a widely held assumption that the brain performs perceptual inference by combining sensory information with prior expectations, weighted by their uncertainty. A distinction can be made between higher- and lower-level priors, which can be manipulated with associative learning and sensory priming, respectively. Here, we simultaneously investigate priming and the differential effect of auditory vs. visual associative cues on visual perception, and we also examine the reliability of individual differences. Healthy individuals (N = 29) performed a perceptual inference task twice with a one-week delay. They reported the perceived direction of motion of dot pairs, which were preceded by a probabilistic visuo-acoustic cue. In 30% of the trials, motion direction was ambiguous, and in half of these trials, the auditory versus the visual cue predicted opposing directions. Cue-stimulus contingency could change every 40 trials. On ambiguous trials where the visual and the auditory cue predicted conflicting directions of motion, participants made more decisions consistent with the prediction of the acoustic cue. Increased predictive processing under stimulus uncertainty was indicated by slower responses to ambiguous (vs. non-ambiguous) stimuli. Furthermore, priming effects were also observed in that perception of ambiguous stimuli was influenced by perceptual decisions on the previous ambiguous and unambiguous trials as well. Critically, behavioural effects had substantial inter-individual variability which showed high test–retest reliability (intraclass correlation coefficient (ICC) > 0.78). Overall, higher-level priors based on auditory (vs. visual) information had greater influence on visual perception, and lower-level priors were also in action. Importantly, we observed large and stable differences in various aspects of task performance. Computational modelling combined with neuroimaging could allow testing hypotheses regarding the potential mechanisms causing these behavioral effects. The reliability of the behavioural differences implicates that such perceptual inference tasks could be valuable tools during large-scale biomarker and neuroimaging studies.

## Introduction

Perception can be understood as an unconscious inferential process that estimates the state of the environment by combining prior expectations with incoming sensory data, weighted by their uncertainty^1–4^: the less informative sensory data becomes, the more observers will rely on their prior expectations that are influenced by their previous experiences^5–8^. Behavioural evidence robustly supports these principles: Ellson were among the first to demonstrate sensory conditioning and induced perception in the absence of sensory stimulation (i.e. conditioned hallucinations) in healthy humans^6^. In addition, Brogden’s sensory pre-conditioning experiment in dogs revealed that associative learning can operate on multisensory representations (also see^9,10^ for more recent reviews of multisensory integration): a light and a sound were repeatedly presented together and by subsequently associating one but not the other stimulus with a shock, the latter still triggered a conditioned response^11^. Furthermore, simple frequency statistics of previous sensory input and percepts, and even internally generated mental images (i.e. imagery) also affect perception^1,12–14^. For instance, repetition priming refers to the well-established phenomenon that exposure to perceptual objects facilitates their subsequent processing^15^ and mental images alone (in the absence of sensory stimulation) can drive the perception of ambiguous stimuli^16,17^. Importantly, the above mentioned phenomena can be derived from the idea that the brain is an organ of inference^4^ that actively optimises a probabilistic model of its environment and uses it to select actions^2,3^.

The neural correlates of perceptual inference have been extensively studied. The influence of prior beliefs on perception is implemented by top-down feedback signals arriving from higher-level multimodal association areas to sensory areas, mirrored by the modulation of posterior alpha by frontal delta^18^ or parietal beta activity^19^. These signals seem to carry information as prior expectations induced by sensory conditioning activate feature-specific stimulus representations in the human primary visual cortex^20^. On the other hand, bottom-up processing of sensory data in unimodal sensory areas (e.g. visual cortex) is assumed to be implemented by gamma-band oscillations^19^. As mentioned above^3^, prior expectations have a stronger weight when sensory input is uncertain and at the neural level, increased frontal theta and fronto-parietal and occipital-parietal beta power have been observed when processing ambiguous stimuli^21^, while the precision of lower-level priors in the primary visual cortex was reduced if the environment was inconsistent^22^. Finally, lower-level priming effects co-occur with reductions in neural activity—termed ‘repetition suppression’^23^ or ‘expectation suppression’^1^—which may reflect top-down effects of expectations based on stimulus frequency statistics^24^.

Critically, there are differences between individuals in terms of the weighting of sensory information vs. prior beliefs during perceptual inference, and evidence suggests that this variation might be of clinical relevance. A number of studies have examined the alterations of perceptual inference in association with psychosis-spectrum conditions or, in line with a continuum approach, with their extended phenotypes in the general population (such as schizotypal traits^25^ or psychotic-like experiences^26^). For instance, delusions may emerge to explain anomalies that arise due to weaker lower-level predictions while stronger higher-level priors might be responsible for the rigidity of delusional beliefs. In an experiment, where participants were shown bistable binocular stimuli, the percept changed more frequently in patients with schizophrenia who experienced delusions and also in healthy participants prone to delusions; furthermore, they were more prone to the effect of expectation manipulations influencing their perception of ambiguous stimuli (wearing ‘special’ glasses)^27,28^. Additionally, hallucinations can be explained by stronger top-down priors overwriting sensory evidence and indeed, voice-hearing individuals (independently of a diagnosable psychotic disorder) are more prone to conditioned hallucinations^7^. In summary, carefully designed paradigms that reliably capture individual differences are needed to provide mechanistic explanations for hallucinations and delusions in the psychosis spectrum.

Here, we build upon the paradigm of Weilnhammer and colleagues^8^ who implemented an audiovisual sensory conditioning task to study the hierarchical neural representation of perceptual states in a changing environment. In their task, motion directions were probabilistically associated with auditory cues. Following the presentation of a cue, participants had to make predictions based on conditional expectations. Then, they made perceptual decisions about disambiguated or ambiguous target stimuli (sensory uncertainty, see^27^). In order to induce associative learning, most trials followed an underlying cue-target association rule (e.g. high tone—counter-clockwise tilt). On some trials, however, the presented tilt violated expectations (e.g. high tone—clockwise tilt) and cue-target contingency changed from time to time (rule uncertainty, see^27^). Computations underlying perceptual decisions were inferred with hierarchical Bayesian models. According to the best fitting model, higher-level predictions based on cue-target contingency (associative learning) had greater weight, relative to lower-level predictions based on previous sensory data (sensory priming). At the neural level, higher-level predictions correlated with activity in the orbitofrontal cortex and the hippocampus, while lower-level predictions correlated with activity in the retinotopic visual cortex.

This elegant study motivates several intriguing research questions that demand careful analysis at the level of behaviour^30^. First, the experiment of Weilnhammer et al. was designed to maximize the effect of associative learning, by explicitly asking participants to report their expectations right after the presentation of the associative cues. One may reason that such subjective reports trigger causal metacognition and explicit memory which may interfere with task performance^9^. To examine interactions between higher- and lower-level prior expectations within the same task, we aimed to increase the effect of sensory priming, therefore, we shortened the time frame available to respond and did not ask participants to make their predictions explicit. We aimed to reduce the influence of conscious, top-down processing by providing a shorter time window for responses, as compared to Weilhammer and colleagues’ study. Following Lawson et al.^31^, we argue that despite the latter modification, data from this adapted design contains sufficient information for the assessment of predictive processes (i.e. these can be inferred from responses and reaction times). Additionally, this way participant burden (i.e. the overall time needed for the experiment) was drastically reduced, which may facilitate data quality. Second, the experimental setting of Weilnhammer et al. consisted of auditory cues and visual targets, thus, associative learning relied on cross-modal associative learning. Using multimodal cues (from both the acoustic and visual domain), we examine whether auditory vs. visual associative cues dominantly influence perceptual decisions about uncertain visual targets. One could reason that if visual sensations are uncertain, sounds will become more important to infer the state of the world, and thus, one may expect the dominance of auditory cues during the perception of ambiguous visual stimuli^3,9^. Last but not least, although individual differences were evident in the data of Weilnhammer et al., no systematic investigation of these were reported in their study. The reliability of behavioural tasks as indicators of individual differences is often overlooked^32,33^ despite the crucial importance of accurate measurement in the study of personality^34,35^ and endophenotypes^36^. Strikingly, despite the growing interest in perceptual inference in clinical neuroscience^37^, to our best knowledge, relatively little information is available on the reliability of indicators derived from perceptual inference tasks. To estimate the reliability of differences in task performance as trait markers, individuals performed our experiment twice.

In summary, our hypotheses were the following: (1) We replicate Weilnhammer and colleagues' findings by showing evidence of higher-level priors affecting task performance (i.e. associative learning effects reflected in accuracy and reaction times), as well as evidence of lower-level priors (i.e. priming effects). (2) We predict that auditory cues dominate perception in ambiguity due to uncertainty in the visual domain (introduced by ambiguous visual targets). (3) Temporally stable individual differences can be measured in a one-week interval. To obtain benchmarks for test–retest stability in the present sample, we also assess schizotypal traits with a questionnaire, which shows excellent reliability. (4) There will be a positive association between higher positive schizotypy and stronger effect of expectations. Furthermore, we explored the associations between perceptual inference and other dimensions of schizotypy.

## Methods

### Participants

Based on an a priori power calculation^38^, we aimed to obtain analysable data from at least 28 participants (expected minimum test–retest effect size r = 0.5, power = 90%, alpha = 0.05). We reasoned that if individual differences in perceptual inference have a meaningful trait-like component, then the one-week test–retest reliability should be at least r = 0.5. Accounting for potential exclusion and dropout, we recruited 34 young adults to take part in the study. Of this initial sample, 2 participants were excluded due to self-reported previous or concurrent psychiatric disorder or any disease affecting the central nervous system. Following inspection of raw data, data of 3 participants from both sessions were excluded because their behaviour suggested that they did not follow the instructions. Furthermore, we excluded the first session data of one participant, and the second session data of another participant, due to their anomalous behaviour (see details in “Results”/Ensuring that participants followed task instructions and generated reliable data). The final sample comprised 29 individuals (13 men, mean (SD) age = 27.8 (9.6) years, mean (SD) education = 17.3 (1.6) years). Participants repeated the experiment after one week. Informed consent was obtained from all subjects. The study was conducted in accordance with the Declaration of Helsinki and it was approved by the United Ethical Review Committee for Research in Psychology (Egyesített Pszichológiai Kutatásetikai Bizottság; EPKEB, 2016/032) that is affiliated with the Department of Cognitive Science, Budapest University of Technology and Economics, and various other institutions conducting psychological/behavioural research (for a complete list of participating institutions, please see http://epkeb.ttk.hu/resztvevok.html).

### Perceptual inference paradigm

**Figure 1:**
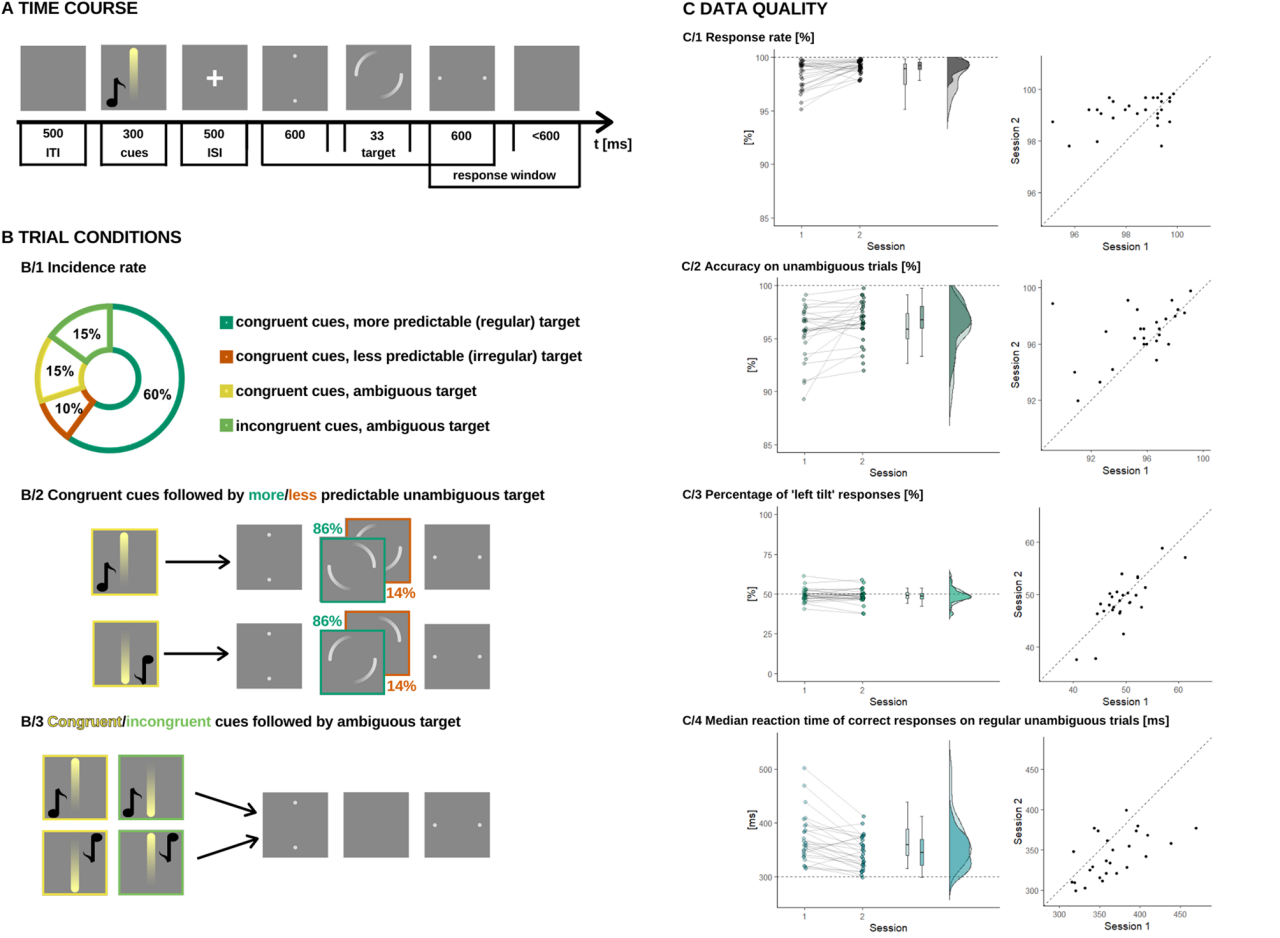
Experimental paradigm and data quality. (**A**) Time course. At the beginning of every trial, a multimodal cue pair was presented for 300 ms consisting of a yellow dot moving up or down and a higher or lower tone. This was followed by a 500 ms interstimulus interval, during which a fixation cross attracted attention to the middle of the screen. Then we presented dot pairs in vertical, then in horizontal position for 600–600 ms. Between these for 33 ms a motion strike indicated a turning illusion, disambiguating the stimuli, or on ambiguous trials we showed nothing (just the gray background), keeping the stimuli bistable. Participants were asked to indicate the perceived direction of the motion, their button presses were detected in the time interval from the presentation of the horizontal dotpair, until maximum 1200 ms later. We used a 500 ms long intertrial interval. (**B**) Trial conditions. Throughout the experiment 30% of the trials were ambiguous (no motion illusion presented). The cues presented were congruent (high tone + top-down movement, low tone + bottom-up movement) when preceding the unambiguous stimuli, and on half of the ambiguous trials. On the other half of the ambiguous trials the cues were incongruent (low tone + top-down movement, high tone + bottom-up movement). Congruent and incongruent cue-pairs remained the same throughout the experiment and across sessions. Trial conditions were balanced in every block of 40 trials. In-block trial order was randomized. In 8 blocks the underlying rule associated 1–1 congruent cuepair with 1–1 (more predictable) direction (0.6 = P(left | high tone, top-down move) = P(right | low tone, bottom-up move) > P(left | low tone, bottom-up move) = P(right | high tone, top-down move) = 0.1), in the other 8 to the other (0.6 = P(right | high tone, top-down move) = P(left | low tone, bottom-up move) > P(right | low tone, bottom-up move) = P(left | high tone, top-down move) = 0.1). These blocks followed each other in random order. (**C**) Data quality. Within each panel of the figure, on the left side, dots represent participants, and the values from each session are connected along the x axis, a boxplot (median, 50% CI) is shown, and the distribution for each session can be seen. While on the right side of the panels, each dot represents a participant's performance in session 1 and 2 on the x and the y axis, respectively. Dashed line indicates the hypothetical perfect correlation. Please find test–retest statistics in Table 1. [C/1] Overall response rate was very high. Percentage of valid responses on 640 trials is presented. Test–retest stability was low, probably due to a ceiling effect. [C/2] Accuracy on unambiguous trials was very high. Percentage of correct answers on 448 unambiguous trials is shown. Test–retest stability was weak, which might be due to ceiling performance. [C/3] There was no general response tendency in the data. Percentage of ‘left tilt’ responses on 640 trials is shown. Test–retest stability was high. [C/4] Processing speed. Median RT of correct responses on 384 regular unambiguous trials. Test–retest stability was high and motor learning was evident.

The paradigm was based on the work of Weilnhammer and colleagues^8^. In this task, participants had to pay attention to vertical point pairs which tilted to a horizontal state and they were asked to indicate the perceived direction of the movement (clockwise or counter-clockwise) with a button press. In our study, participants most probably were shown a tilting illusion which made the percept unambiguous but on 30% of the trials, the stimulus was ambiguous and only the initial (vertical) and the final (horizontal) state were shown (see Fig. 1B). Before every tilt, multimodal cues were presented which consisted of a higher (576 Hz) or lower (352 Hz) sound and a yellow disk moving upwards or downwards. On unambiguous trials, the cues were congruent in that the acoustic and the visual cue always predicted the same movement direction. In order to facilitate learning of the underlying rule, on 86% of unambiguous trials (60% of all trials), congruent cues were followed by one type of tilt (*regular* trials). However, on 14% of unambiguous trials (10% of all trials), congruent cues were followed by a tilt in the opposing direction (*irregular* trials). For example, if on regular unambiguous trials a lower pitch and an upwards moving yellow disk were followed by a clockwise tilt, then, on irregular unambiguous trials, these cues were followed by a counter-clockwise tilt. Furthermore, on one half of the ambiguous trials, the cues were congruent (auditory and visual cues predict the same tilt), while on the other half, incongruent (auditory and visual cues predict opposite tilts) cues were presented. Congruent cue-pairs remained the same (higher pitch and downwards movement, lower pitch and upwards movement) throughout the experiment and across sessions.

The task included 640 trials which were presented in 16 randomly ordered consecutive blocks, without any break. The probability distribution of conditions was balanced in each block of 40 trials and during the entire experiment too. The probabilistic rule predicting one particular direction (e.g. combination of high pitch and downward movement before a clockwise tilt) could change from block to block (e.g. high pitch and downward movement before a counter-clockwise tilt). Participants were informed that the cues are related to the direction of the motion, but the association between cues and motions may change from time to time.

### Procedure

The experiment lasted about 30 min and was performed in a room with closed windows, artificially and evenly lit. Participants listened to the auditory stimuli through headphones. Visual stimuli were presented on a computer screen (generic PnP monitor) with 60 Hz refresh rate. The task was built in and presented with Psychopy 3.2.3. under Python v3.7. First, we estimated hearing thresholds (using 464 Hz sounds with varying volume [10 levels] in a randomly presented order) in order to individually ensure the audibility of the acoustic cues. Then, participants were trained to appropriately respond to the tilting directions (clockwise tilt—right arrow button, counterclockwise tilt—left arrow button) as fast as possible, but at maximum within 1200 ms. Please note that we aimed to reduce the influence of conscious, top-down processing by providing a shorter time window for responses, as compared to Weilhammer et al. (For task timing information see Fig. 1A.) After the perceptual inference paradigm, participants were asked to complete a short questionnaire that assessed schizotypal traits.

### Schizotypal traits

We were interested to see whether objectively assessed differences in perceptual inference are associated with self-reported variation in perception and cognition. Therefore, we measured positive, negative and disorganized dimensions of schizotypal traits (that is, personality traits resembling the signs and symptoms of schizophrenia) with the Oxford–Liverpool Inventory of Feelings and Experiences, short version (sO-LIFE^39^, Hungarian version^40^). An additional purpose of administering this questionnaire was to obtain benchmarks for test–retest stability in the present sample with an instrument optimized for the assessment of individual differences. Here, we used three subscales. Positive schizotypy was measured with the Unusual Experiences subscale (12 items, reliability in the sample Cronbach’s alpha = 0.82/0.82, test–retest reliability Spearman’s rho = 0.92, ICC = 0.96 ), negative schizotypy was measured with the Introvertive Anhedonia subscale (10 items, reliability in the sample Cronbach’s alpha = 0.76/0.74, test–retest reliability Spearman’s rho = 0.86, ICC = 0.94), and disorganized schizotypy was assessed with the Cognitive Disorganisation subscale (11 items, reliability in the sample Cronbach’s alpha = 0.78/0.84, test–retest reliability Spearman’s rho = 0.87, ICC = 0.95). Studies have shown that the sO-LIFE has appropriate psychometric properties to assess schizotypal traits in the non-clinical population (except for the Impulsive Nonconformity dimension, which we therefore did not use in our study, reliability in the sample Cronbach’s alpha = 0.74/0.70, test–retest reliability Spearman’s rho = 0.44, ICC = 0.91)^41^. Although the original questionnaire uses a binary response format, we decided on 5-level Likert-format in order to improve reliability (following^40^), which was excellent in the present sample. Additional data (mean, SD, minimum, maximum, skewness, kurtosis) about these subscales can be found in Supplement 1.

### Statistical analyses

First, in order to ensure data quality, we explored raw data to identify potential anomalies in task performance (i.e. overall response rate and reaction times, accuracy on congruent regular unambiguous trials, severe response bias on all and/or ambiguous trials), and we excluded participants who suspectedly did not follow task instructions (see “Participants” section).

Then, we examined the effects of higher- and lower-level predictions on perceptual inference. The overall effect of higher-level predictions (sensory conditioning/associative learning) is reflected in perceptual decisions being consistent with the congruent cues on ambiguous trials. Moreover, we argued that if participants learned the associative rule, their responses on irregular unambiguous trials should be slower and less accurate. Critically, performance on ambiguous trials where the cues are incongruent allow the examination of unisensory vs. multisensory higher-level predictions: perceptual decisions consistent with the acoustic (vs. visual) cue indicate multisensory (vs. unisensory) dominance. Furthermore, lower-level predictions are mirrored in perceptual decisions consistent with the stimulus presented on the previous unambiguous trial (sensory priming) and with the perceptual decision on the previous ambiguous trial (perceptual priming). The above experimental effects were analysed with nonparametric Wilcoxon t-tests and *r* (= *Z*/sqrt(*N*)) was computed as a measure of effect size.

Finally, test–retest reliability was analysed with Spearman’s rank order correlations and intraclass correlations (ICC(3,k), based on two-way mixed effects model, aiming for consistency in k = 2 measures, following^33^). Visualisations were created following the excellent work of van Langen^43^.

Analyses were performed with R^44^ and the R-packages *broom*^45^, *carData*^46^, *corrplot*^47^, *effects*^48^, *effsize*^49^, *factoextra*^50^, *forcats*^51^, *Formula*^52^, *GGally*^53^, *ggcorrplot*^54^, *gghalves*^55^, *ggstatsplot*^56^, *gridExtra*^57^, *Hmisc*^58^, *knitr*^59^, *lattice*^60^, *papaja*^61^, *psych*^62^, *readr*^63^, *readxl*^64^, *rmarkdown*^65^, *survival*^66^, *tidystats*^67^, and *tidyverse*^68^.

## Results

### Ensuring that participants followed task instructions and generated reliable data

After the inspection of raw data, we decided to exclude data of 2 participants because their behaviour suggested that they did not follow the instructions (they had disproportionately fast responses on unambiguous trials (*median* RT < 230 ms vs. *median* RT [*IQR*] in the whole sample = 352 [323; 378] ms), and we also excluded another participant’s data who at debriefing reported that they purposely adopted a strategy to respond ‘left’ on ambiguous trials, which was also reflected in their behaviour: proportion of ‘left’ responses on ambiguous trials = 91%). Furthermore, we excluded data from two participants from the first and the second session, respectively, due to their anomalous behaviour (one had a 77% response rate on all trials during the first session, while *median* [*IQR*] response rate in the whole sample = 99 [98; 99] %]; and the other had an 83% accuracy on trials where performance should be near 100%, as expected unambiguous stimuli are shown: *median* [*IQR*] accuracy on these trials in the whole sample = 97^93,97^% ).

In the final sample, response rate (*mean (SD)* = 99.2 (1.07) %) and accuracy (*mean (SD)* = 96.5 (2.26)%) for unambiguous stimuli were very high, indicating that participants followed task instructions (see Fig. 1C/2,3). Then, we evaluated whether there was any indication of response tendency. Proportion of ‘left’ responses did not differ from 50%, as calculated with one-sample Wilcoxon T-test, which is perfectly reasonable given the balanced design of the task and high accuracy for unambiguous stimuli (See Fig. 1C/3 and Table 1. GenRespTend[%]). Finally, we inspected cognitive-perceptual processing speed. We calculated participants’ median RT of accurate responses on regular unambiguous trials (*median (SD)* = 370 (39) ms). Motor learning was indicated by shorter reaction times in the second session, relative to the first session (see Fig. 1C/4).

**Table 1:**
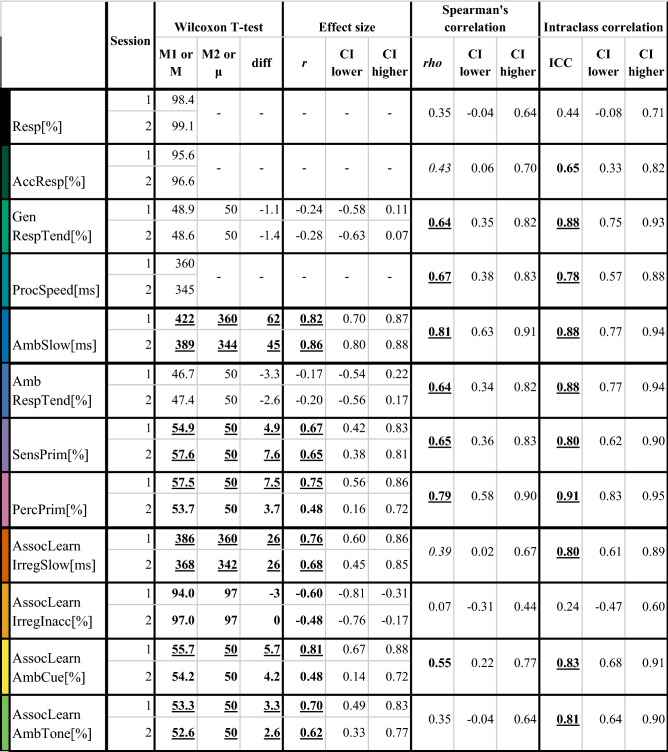
Group level tendencies. Individual differences in task performance have high temporal stability over a 1-week interval.

In the first group of columns, the results of paired Wilcoxon T-tests results are shown, with the null hypothesis being that populations’ mean ranks are equal (*N* = 27). In the case of one sample comparisons (*N* = 29), we tested the null hypothesis that the median is equal to a theoretical value, namely 50% (as chance level).

In the middle, we show the effect size of the difference (*r* = Z/sqrt[N]) and its 95% confidence interval. In the last group of columns we show Spearman’s rank order correlation coefficients (rho), intraclass correlation coefficients (ICC3,k) and their 95% confidence intervals (*N* = 27; *df* = 26).

In a frequentist framework, a 95%CI should be interpreted as follows: if the true value is smaller (larger) than the lower (upper) bound, only 2.5% of the time would we expect a test–retest correlation as large (small) as we have found ^70^. Thus, true values of 1-week test–retest reliability outside these intervals are contradicted by the data (with an error rate of 5%).

Variable labels: Resp[%]. Response rate (%); AccResp[%]. Accurate responses under certainty (% of correct responses); GenRespTend[%]. General response tendency (% of ‘left’ response); ProcSpeed[RT]. General perceptual speed (RT to expected certain stimuli, ms); AmbSlow[RT]. Perceptual inference speed under uncertainty (RT to unexpected minus expected, ms); AmbRespTend[%]. Response tendency under uncertainty (% of ‘left’ response); SensPriming[%]. Sensory priming effect under uncertainty (% consistent with perceptual decision on previous unambiguous trial); PercPriming[%]. Perceptual decision priming effect under uncertainty (% consistent with perceptual decision on previous ambiguous trial); AssocLearnIrregSlow[RT]. Associative learning effect under certainty—cost of irregularity (ms RT); AssocLearnIrregInacc[%]. Associative learning effect under certainty—cost of irregularity (% accurate responses); AssocLearnAmbCue[%]. Associative learning effect under uncertainty—cue-consistency (congruent cues); AssocLearnAmbAuditory[%.] Associative learning effect under uncertainty—auditory dominance (incongruent cues).

*p* < 0.05; *p* < 0.01; *p* < 0.001.

### Ambiguous stimuli take more time to process

**Figure 2:**
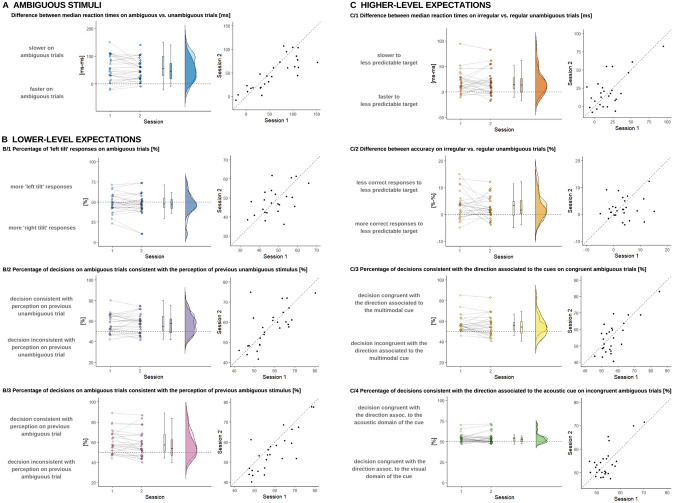
Processing of ambiguous stimuli and the influence of lower- and higher-level expectations. Within each panel of the figure, on the left side, dots represent participants, and the values from each session are connected along the x axis, a boxplot (median, 50% CI) is shown, and the distribution for each session can be seen. While on the right side of the panels, each dot represents a participant's performance in session 1 and 2 on the x and the y axis, respectively. Dashed line indicates the hypothetical perfect correlation. Please find test–retest statistics in Table 1. (**A**) Processing of ambiguous stimuli takes more time. On the y axis, median reaction times differences on unambiguous-ambiguous trials (the target stimuli was made unambiguous with motion streaks). If there was no difference between these conditions, dots should be around 0 ms (level shown with dashed line). Test–retest stability was high and general motor learning was also observed. (**B**) Effects of lower-level priors captured by response tendency on ambiguous trials. [B/1] Response tendency in ambiguity. Along the y axis, we present the percentage of ‘left’ decisions in ambiguity. If there were no tendency, participants should perform around chance level (50%, shown with dashed line). Test–retest stability was high. [B/2] Sensory priming effect in ambiguity. Along the y axis, we present the percent of decisions consistent with the previous decision made in unambiguity. If there were no sensory priming, participants should perform around chance level (50%, shown with dashed line). Test–retest stability was high. [B/3] Effect of perceptual priming in ambiguity. Along the y axis, we present the percentage of decisions consistent with the previous decision made in ambiguity. If there were no perceptual priming, participants should perform around chance level (50%, shown with dashed line). Test–retest stability was high. (**C**) Effects of higher-level priors captured by reaction time and accuracy on unambiguous trials and by response tendency on ambiguous trials. [C/1] Slowing down to unexpected (vs. expected) targets. On the y axis, median reaction times differences between irregular—regular unambiguous trials (when cues were followed by less vs more predictable tilting illusion according to the probabilistic rule). If there was no difference between these conditions, dots should lie around 0 ms (level shown with dashed line). Test–retest stability was high and motor learning was indicated by shorter reaction times in session 2. [C/2] Decreased accuracy to unexpected vs. expected targets. On the y axis, the difference between the % of correct responses to irregular—regular unambiguous stimuli are presented. If there was no difference between these conditions, dots should be around 0 ms (level shown with dashed line). Test–retest stability was poor, probably due to a ceiling effect. [C/3] Cues influence responses to ambiguous targets. Along the y axis, we present the percentage of decisions consistent with the direction associated with the cues in ambiguity when the cues were congruent. If there were no effect of associative learning on perceptual decisions, participants should perform around chance level (50%, shown with dashed line). Test–retest stability was high. [C/4] Auditory cues dominantly influence responses to ambiguous targets. Along the y axis, we present the percentage of decisions consistent with the direction associated to the acoustic cue in *ambiguity* after *incongruent* cues (where the acoustic and visual cues were associated with different directions). If there were no dominance of any modality, participants should perform around chance level (50%, shown with dashed line). Test–retest stability was high.

To measure the effect of stimulus uncertainty, we performed Wilcoxon T-tests comparing median reaction times on unambiguous vs. ambiguous trials (target stimuli containing visible tilting illusion or not). We found significant differences in both sessions. Reaction times were significantly higher on ambiguous than on unambiguous trials, suggesting slower processing of uncertain stimuli (see Fig. 2A and Table 1. AmbSlow[ms]).

### Lower-level priors effects influence perception of ambiguous stimuli—evidence of sensory and motor priming

We examined three indicators of lower-level priors. First, we considered the priming effects of sensory data encountered on the previous unambiguous trial. Second, we evaluated the influence of the preceding perceptual inference under uncertainty on the previous ambiguous trial. Third, we examined whether participants demonstrated motor memory/priming as reflected in their tendency to repeat a certain response (this can also be interpreted as simple response/decision tendency). These are represented by the perceived tilting direction on the previous unambiguous trial, decisions made on the previous ambiguous trial and tendency towards a certain response (independent of experimental manipulation), respectively. We performed one-sample Wilcoxon T-tests to measure the effect of lower level priors (i.e. sensory priming) on ambiguous trials (see Table 1. AmbRespTend[%], SensPrim[%] and PercPrim[%]). The proportion of decisions participants made on ambiguous trials consistent with the decision they made on the previous ambiguous trial was significantly higher than what would be expected by chance (μ = 50%). In addition, the proportion of decisions on ambiguous trials consistent with the decision on the previous unambiguous trial was also significantly higher than chance level (μ = 50%). We found no significant general response tendency towards any direction at the group level, but there was large variability across individuals (see Fig. 2B/1–3.).

### Higher-level priors based on associative learning also influence perceptual inference—evidence of sensory conditioning

We performed several Wilcoxon T-tests to investigate the effect of associative learning. First, we compared median reaction times on regular vs. irregular unambiguous trials (when cues were followed by a more vs. less predictable tilt) with paired Wilcoxon T-tests in each session and found that participants reacted slower on irregular, than on regular trials (see Fig. 2C/1 and Table 1. AssocLearnIrregSlow[ms]). Second, we compared the rate of correct responses on regular vs. irregular unambiguous trials in each session and found that participants made more errors on irregular trials than on regular trials (see Fig. 2C/2 and Table 1. AssocLearnIrregInacc[%]). Third, we performed one-sample Wilcoxon T-tests to measure if decisions differed from chance level (μ = 50%) on ambiguous trials when cues were congruent. We found that participants responded more consistently with the direction associated with the cues (See Fig. 2C/3 and Table 1. AssocLearnAmbCue[%]). These results suggest that participants developed expectations (higher-level priors) consistent with the cues’ prediction regarding tilting direction, and this resulted in slower responses and more errors when the expectation was violated by the presented tilt, relative to when their expectations were met by the stimulus. Furthermore, these expectations shaped their perceptual decisions when they were faced with ambiguous target stimuli.

### When the cues are conflicting, auditory cues dominate the perception of ambiguous visual stimuli

To examine whether the modality of the cues caused behavioral differences in participants, we focused on those trials where after an incongruent cue ambiguous target was presented, because here the cues from the acoustic and the visual domain were associated with different directions. We performed a one-sample Wilcoxon T-test, comparing chance level (μ = 50%) to the proportions of decisions consistent with auditory cues in both sessions, on *incongruent ambiguous* trials. Importantly, we found that decisions were significantly more likely to be in line with the direction associated with the acoustic cue (see Table 1. AssocLearnAmbTone[%] and Fig. 2C/4.).

### Individual differences in behavioural effects have robust test–retest stability

All the above effects were relatively subtle at the group level. However, we observed substantial variation between individuals in terms of the magnitude of these effects. Now, we turn to evaluate whether these reflect temporally stable, trait-like differences between participants. To measure the reliability of our metrics, we performed a test–retest analysis using Spearman’s rank correlation across sessions, which tests whether the ranking of individuals within the group was similar at the two sessions for a given variable. Furthermore, we calculated the intraclass correlation coefficient (ICC), which reflects both the degree of correlation and the agreement between measurements (see details in Table 1 and in the scatterplots in the right side of Figs. 1 and 2).

First of all, cognitive-perceptual processing speed was reliable, which allowed further testing of reaction time differences. Slowing down on ambiguous (vs. unambiguous) trials had high test–retest reliability. This suggests that although participants demonstrated motor learning (i.e. they became faster) throughout the experiment, in-group ranks stayed relatively consistent.

Indicators of lower-level priors were based on response tendencies deviating from chance level (50%) towards consistency with previously made perceptual decisions. As can be seen from the histograms (Fig. 2B), the extent of these effects varied greatly between participants. Importantly, this variation across individuals in sensory and perceptual priming were both stable over the test–retest interval. When examining motor memory, we did not find a tendency towards any direction at the group level (neither on all trials nor in ambiguity), but individual scores varied highly here too. These differences were stable throughout the sessions, as shown by high test–retest *rho* and ICC values.

Variables reflecting associative learning (see Fig. 2C) were calculated in multiple ways. First, on unambiguous trials, we examined the behavioural effects of expectation violation. Differences in the extent of slowing down on irregular (vs. regular) trials was reliable, but differences in the degree of reduced accuracy were not. Second, we examined response tendencies on ambiguous trials. The proportion of cue-consistent decisions were reliable, and so was the dominance of the acoustic domain on trials with incongruent cues.

Interestingly, individual differences in the behavioural effects of expectation violation and in the measures of attention were reliably captured by reaction time but not by decision accuracy, further strengthening our rationale that reaction times not only carry important information but they may provide a more sensitive form of measurement. Low reliabilities were observed for average response rate and weakened accuracy of responses on irregular unambiguous trials, which could be explained by almost perfect performance (i.e. ceiling effects) resulting in low and uninformative variability.

Visual inspection of the scatterplots suggested that outliers might bias the estimation of test–retest reliability. Thus, we recalculated the test–retest rho and ICC values for variables where prominent outliers were found. First, after exclusion of a participant from the extent of slowing down on irregular (vs. regular) trials (AssocLearnIrregSlow[ms], top right corner in Fig. 2C/1), reliability estimates based on Spearman's correlations were no longer significant (rho = 0.32[− 0.08, 0.63], *p* = 0.11), while the intraclass correlation coefficient reduced to fair (ICC = 0.63[0.27, 0.81], *p* < 0.01). Second, exclusion of the most extreme participant in the proportion of cue-consistent decisions on ambiguous trials (AssocLearnAmbCue[%], top right in Fig. 2C/3) did not influence test–retest reliability (Spearman’s test–retest rho = 0.5[0.14, 0.74], *p* = 0.01; ICC = 0.7[0.42, 0.85], *p* < 0.01). Third, exclusion of the two most extreme points in the proportion of acoustic-cue consistent decisions on ambiguous trials (AssocLearnAmbTone[%], top right in Fig. 2C/4) resulted in insignificant Spearman’s test–retest and intraclass correlation coefficients (rho = 0.18[− 0.23, 0.54], *p* = 0.39; ICC = 0.39[− 0.22, 0.69], *p* = 0.119). Nonetheless, it should be kept in mind that these outliers passed our behaviourally defined exclusion criteria (see “Methods”), and their performance was consistent across the two sessions, suggesting that their extreme scores reflect true variance. By excluding these outliers, individual variation in the sample was reduced, which may have limited the statistical power of the test–retest reliability analyses.

### Exploratory analyses: internal structure of perceptual inference indicators and their relationship with schizotypal traits

**Figure 3:**
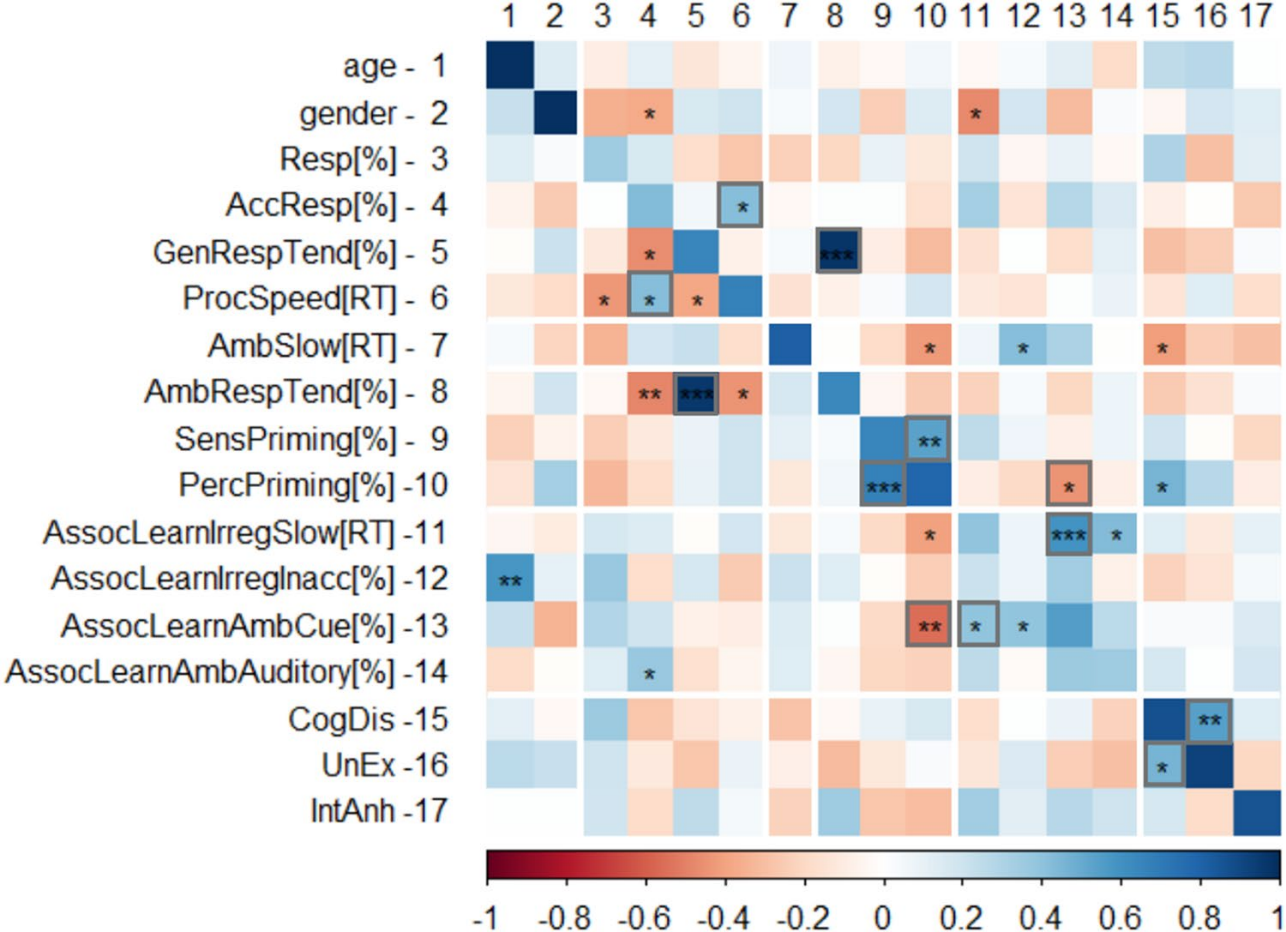
Correlations between task performance, schizotypal traits and demographics. Spearman’s rank order correlation of each variable. Lower triangle: session 1, upper triangle: session 2, diagonal: test–retest. Significance levels: *: *p* < 0.05, **: *p* < 0.01, ***: *p* < 0.001. Grey rectangles indicate replicated significant correlations (both sessions). Variable labels: 1: age; 2: gender (higher [blue] = males score higher); 3: Resp[%]. Response rate (%); 4. AccResp[%]. Accurate responses under certainty (% of correct responses); 5. GenRespTend[%]. General response tendency (% of ‘left’ response); 6. ProcSpeed[RT]. General perceptual speed (RT to expected certain stimuli, ms); 7. AmbSlow[RT]. Perceptual inference speed under uncertainty (RT to unexpected minus expected, ms); 8. AmbRespTend[%]. Response tendency under uncertainty (% of ‘left’ response); 9. SensPriming[%]. Sensory priming effect under uncertainty (% consistent with perceptual decision on previous unambiguous trial); 10. PercPriming[%]. Perceptual decision priming effect under uncertainty (% consistent with perceptual decision on previous ambiguous trial); 11. AssocLearnIrregSlow[RT]. Associative learning effect under certainty—cost of irregularity (ms RT); 12. AssocLearnIrregInacc[%]. Associative learning effect under certainty—cost of irregularity (% accurate responses); 13. AssocLearnAmbCue[%]. Associative learning effect under uncertainty—cue-consistency (congruent cues); 14. AssocLearnAmbAuditory[%.] Associative learning effect under uncertainty—auditory dominance (incongruent cues); 15. CogDis. Cognitive disorganisation—O-Life subscale sum; 16. UnEx. Unusual experiences—O-Life subscale sum; IntAnh. Introvertive anhedonia—O-Life subscale sum.

We conducted Spearman’s rank order correlation analyses using age, gender, task-derived indicators and self-reported alterations of perception and cognition (i.e. schizotypal traits) (see Fig. 3). Given that our sample is rather low in the context of cross-method individual difference research^70,71^, these analyses are highly exploratory, essentially no non-trivial correlation remains significant after FDR adjustment of *p* values; nevertheless, if consistent patterns emerge across sessions, it may facilitate the generation of hypotheses.

First, we predicted positive correlations between measures of higher-level priors (the extent of slowing down and increased error rate to less predictable unambiguous targets, the proportion of cue-consistent decisions on ambiguous trials, and the proportion of decisions consistent with the acoustic cue on ambiguous trials where the cues were conflicting). By the look of the heatmap (Fig. 3. #11–14), this tendency is evident but only some of the associations are significant (cue-consistency in ambiguity and slowing down to unexpectedness positively correlated in both sessions). These variables seemed to positively correlate with the extent of slowing down when faced with ambiguous (vs. unambiguous) targets (Fig. 3. #7), although only one of the associations was significant (increased error rate to less predictable targets correlated positively with slowing down on ambiguous trials). Additionally, we observed positive correlations between some of the indicators of lower-level priors (Fig. 3. #8–10): sensory and perceptual priming (proportion of decisions on ambiguous trials consistent with the decision on the previous unambiguous and ambiguous trial, respectively) were positively and significantly associated across individuals.

Furthermore, it is noticeable that these two sets of variables (that putatively indicate the effects of higher- vs. lower-level priors [Fig. 3. #11–14 and #8–10, respectively]) were weakly and negatively correlated with each other. There was a particularly strong and consistent significant negative association between perceptual priming and the effect of congruent cues on perceptual decisions in ambiguity, suggesting that across individuals, the effect of lower- and higher-level expectations might be complementary.

Although we considered response tendency in ambiguity (Fig. 3. #8) as an additional form of lower-level priors, it was not related neither to sensory nor to perceptual priming. Instead, a tendency towards ‘left’ responses on ambiguous trials had a trivial association with general response tendency (Fig. 3. #8 and #6). Neither age or gender demonstrated any consistent and robust association with individual differences in task performance.

Finally, disorganised schizotypy was significantly and negatively related to slowing down to ambiguous, relative to unambiguous stimuli in the second session (Fig. 3. #15 and #7), which may suggest a general tendency to rely more on expectations and less on sensory input. Additionally, disorganisation was significantly and positively associated with perceptual priming in the second session (Fig. 3. #15 and #10). In contrast, negative schizotypy was positively associated with indicators of higher-level priors (based on associative learning) while it was negatively linked to indicators of lower-level priors (sensory and perceptual priming) (Fig. 3. #17 and #11–14, #9–10, respectively). We note that although these associations were not significant, they were consistent across the two sessions.

## Discussion

Perception can be seen as an unconscious inference that combines sensory input with prior expectations in an uncertainty-weighted manner: when input is impoverished, prior knowledge of the observer will play a greater role in determining what is perceived^1–3^. Such prior expectations may have multiple sources^1^: one may distinguish between lower-level expectations based on frequency statistics of the environment (e.g. in general, some stimuli might be more common than others) and higher-level expectations based on conditional probabilities (e.g. some stimuli are more likely to occur in certain temporal or spatial contexts). In the present study, we demonstrated within a single experiment that perception of ambiguous visual stimuli in a changing, multimodal setting is simultaneously influenced by higher- and lower-level prior expectations. Interestingly, when higher-level priors from different modalities were conflicting, auditory (as compared to visual) cues had a stronger influence on the perception of ambiguous visual stimuli. There was substantial variability across individuals in behavioural performance and this variation was temporally stable. This suggests that such perceptual inference tasks might be suitable tools for large-scale biomarker studies^36^ and personality neuroscience research^34,35^.

Our study adds to the growing knowledge about how different expectations shape perceptual decisions under ambiguity. Here, building upon an elegant experimental paradigm^8^, we found that auditory information had a dominant influence on perception of ambiguous stimuli when the cues were conflicting. How can we explain the dominance of the auditory cues, standing in contrast with previous findings of visual dominance in adults, such as the Colavita Dominance Effect^72^? In Colavita’s task, participants were asked to indicate the perceived modality of an auditory or a visual stimuli (a simple tone or a light) with a button press. When presented with bimodal stimuli, participants had a tendency to report perceiving the visual modality, and many did not realise at all that a tone was also presented. Some explained these findings in terms of attentional strategies: as acoustic stimuli are transient, they automatically grab attention, and as a compensatory strategy, humans may allocate more attentional resources to visual stimuli^73^. However, attentional manipulations mostly failed to reverse visual dominance to auditory dominance in adults^72,74,75^. Interestingly, children until the age of 6 show auditory dominance if visual stimuli are more complex, and their bias is not reversible with attentional instructions^76^. Others argue that visual dominance might derive from response bias^73^, as in tasks where response keys are independent of modalities, auditory dominance can be seen in reaction times^77^. In an eye-tracking study, Dunifon et al. found that auditory inference is happening early in the course of processing, and they provided evidence for mixed dominance: while accuracy points to visual dominance, slower responding and delayed latency of first fixation suggest auditory dominance^75^. Indeed, even in Colavita’s original study, participants reacted slower to multimodal targets than to unimodal (visual or acoustic) ones^72^. The most promising efforts made towards findings of auditory dominance (or at least attenuating the visual dominance effects) were manipulations of target frequencies^74,76,78^, suggesting that implicit learning of environmental conditions—adjusting lower-level expectations—does have an effect on modality dominance. In our task, the frequency of visual and auditory cues was balanced, but the uncertainty of the visual domain may have shaped expectations in such ways that visual dominance reversed to auditory. This theory is in line with the *Modality precision hypothesis* which states that when information about some characteristics of an event coming from two sensory modalities are contradictory, the resolution will favor the modality that is more precise of the two in registering that event^79^. We argue that the representation of the overall precision of the visual modality might be reduced by the presence of ambiguous visual targets, thereby ultimately downweighting the salience of visual information in learning and predictive processing^3^. Indeed, unreliable feedback reduces accuracy and deteriorates representation of visual stimuli in the visual cortex^22^. Relatedly, it has been shown that modality dominance effects depend on the strength of the signals, for example in a spatial localization task, where usually visual stimuli dominate (‘vision captures sound’), for severely blurred stimuli the relatively more precise acoustic modality becomes dominant (‘sound captures vision’), explained by optimal combination of both information, based on signal-to-noise ratio^80^. Although here we did not manipulate stimulus intensity or the amount of noise, ambiguity in the visual modality still may have compromised processing of visual information throughout the task. Although the *Modality appropriateness hypothesis* would predict that in a visuospatial task, the visual information should be more appropriate^79^, it also states that auditory information is more appropriate for the perception of temporally structured events, thus, in respect to the relatively fast and monotonous (temporally structured) nature of the task, the acoustic information might be accentuated. Furthermore, one may argue that an imbalance in stimulus magnitude stands behind these results, but as seen in Colavita’s Experiment II^72^, increasing the subjective intensity of the auditory stimulus even by a factor of two did not lead to reversed dominance.

Furthermore, we show that perceptual decisions are comparably influenced by expectations of multiple origin (see effect sizes in Table 1). How such different expectations interact to shape perception and imagery is an outstanding question in cognitive science^1,14^. We found that sensation and perception on the preceding unambiguous and ambiguous trial, respectively, both had an impact on perception of subsequent ambiguous stimuli. It has been argued that such forms of expectations may rely on relatively simple computations implemented within sensory areas (in this case, the visual system)^1^. In addition, we demonstrated that multisensory associative learning also influenced the perception of ambiguous stimuli, and such audio-visual integration is likely to be implemented by higher-order multimodal brain regions^1^. Relatedly, Weilnhammer and colleagues^8^ have found that lower-level expectations correlated with activation in the retinotopic visual cortex, while higher-level expectations correlated with activation in the orbitofrontal cortex and the hippocampus. It has been suggested that the orbitofrontal cortex is anatomically well-suited to encode the actual task state with high complexity^81^. Although state representations encoded in the activation patterns of lower-order sensory areas can be utilised by more elementary reinforcement learning or decision making mechanisms, more complex representations and their integration in the higher-order orbitofrontal cortex might support finely-tuned learning and behavioural adaptation^81^. In addition, pattern completion in the hippocampus may support the reinstatement of activation patterns representing the expected visual stimuli in the early visual cortex^82^.

Individual differences in perceptual inference were highly stable over a one-week interval (median ICC = 0.83, range 0.80–0.91, range of 95%CIs 0.61–0.95). The only exception was the increase in error rate when congruent cues were followed by an unexpected unambiguous stimulus (ICC = 0.24), however, this is not that surprising given that the increase in errors for unexpected vs. expected unambiguous stimuli was minuscule (3% and 0% for session 1 and 2, respectively), in line with the notion that expectations are less likely to influence perceptual decisions when stimuli are certain^1^. Even well-established cognitive paradigms may have poor reliability due to a critical trade-off affecting their design^83^: differences between participants increase noise in the data when effects of experimental manipulations are of interest^84^. Crucially, reliability values in our task are comparable to the highest literature standards for indicators derived from single tasks. Similarly high reliabilities have been documented for oculomotor control (mean ICC of the most reliable indicators from the antisaccade and the smooth pursuit tasks = 0.77, antisaccade error rate ICC = 0.90^75,76^), visual (mean r = 0.76^77^, verbal (r’s range 0.52–0.81)^78^, and complex working memory capacity (r’s range 0.67–0.81^79^) or response inhibition and interference control (mean ICC = 0.74^76^). Although we observed somewhat higher test–retest stabilities for the self-report schizotypy scores (ICC’s range 0.94–0.96, also see the diagonal in Fig. 3), the reliability of the performance-based indicators of perceptual inference in our study is even comparable to that of self-report measures of self-regulation (ICC = 0.67^31^). Importantly, our findings stand in strong contrast with the poor reliability of behavioural measures of self-regulation (median ICC = 0.31, stop-signal reaction time ICC = 0.03^31,76^, implicit learning of statistical structures in the perceptual, cognitive and motor domains (mean r = 0.32^80^, and task-related brain activation (mean ICC = 0.4^81^). These contrasting findings highlight how essential it is to establish the reliability of behavioural tasks prior to their application in clinical and personality neuroscience^34–36^. Strikingly, to our best knowledge, relatively little information is available on the reliability of indicators derived from perceptual inference tasks, despite the growing interest towards them in clinical neuroscience^37^. Although simulations can be used to estimate the accuracy of parameter recovery with a given number of trials (i.e. internal consistency reliability within a single session)^92^, they are uninformative whether the indicator captures a temporally stable, trait-like feature. Nevertheless, some data suggested that individual differences in perceptual inference can be reliably measured. For instance, a behavioural measure of visual imagery strength has been found to have outstanding test–retest reliability (r = 0.86 over 2 weeks)^16^. Others have shown that individuals are characterised by a unique profile of switching between percepts of multistable auditory stimuli and that switching patterns correlate not only with cognitive control and ego resiliency (a personality trait reflecting flexible adaptation)^93^ but also with glutamate–glutamine (Glx) and gamma-aminobutyric acid (GABA) concentrations in sensory and frontal areas, respectively^94^. Although these preliminary findings are encouraging and perceptual inference tasks seem to capture variation relevant to the psychosis-spectrum^37^, lack of knowledge about the reliability of the measures and the stability of individual differences may limit the progress of this promising avenue of research. Thus, future studies should assess the temporal stability of perceptual inference and its neural correlates over longer intervals and in other populations. Reaction times on unambiguous trials where target stimuli are unambiguous were also highly reliable. This variable may reflect processing speed, a core aspect of intelligence^95^. A study has found that perceptual speed was specifically related to increased task-related activation in various visual areas, the left thalamus and precentral gyrus, and the right insula, while it predicted decreased activation in the precuneus, the insula, the angular gyrus and some frontal and temporal areas^96^. Others have shown that white matter integrity in the parietal and temporal lobes bilaterally and in the left middle frontal gyrus predicted processing speed^97^. We speculate that differences in perceptual speed in our task might covary with neural indicators of processing efficiency in the visual and the motor system and in their efficient top-down control by the dorsal attentional system^98^. Establishing stability in clinical individuals would be crucial prior to the application of the paradigm in longitudinal observational and intervention studies.

Drawing on brain imaging studies, one may formulate hypotheses about the potential neurobiological correlates of our behavioural findings. There is evidence that perceptual decisions in ambiguity are governed by neural representations in the primary visual cortex: seeing the first element of a learned sequence of moving dots induces retinotopically specific but temporally compressed replaying of visual sequences in the early visual cortex, which may cause the experience of illusory motion^99^. Relatedly, imagery strength is negatively related to the surface of the primary and the secondary visual cortices, while imagery precision shows an opposite association^16^. As imagery strength may reflect strong influence of priors while imagery precision could indicate increased sensory precision (i.e. likelihood) (see^13^), we are tempted to speculate that stronger influence of higher-level priors in our task might be caused by a smaller surface of the primary and secondary visual cortices. Furthermore, sensory priming, mirroring the effect of lower-level priors, might be mediated by the activation of sensory representations in visual areas, which can occur even in the absence of sensory stimulation^1^. For instance, in individuals with the ‘visual snow’ syndrome (who repeatedly see miniscule, flickering dots), hypermetabolism of the right lingual gyrus and the left cerebellar anterior lobe has been reported^100^. Interestingly, such individuals also report various additional lower-lever, non-meaningful perceptual experiences (e.g. seeing afterimages or photopsia). Another study suggests that the surface area of the primary visual cortex predicts the distinctiveness of subtle differences in orientation, but it is negatively related to modulation by the global context^101^. Based on the above studies, we predict that individuals in whom higher-level priors largely influenced perception of ambiguous stimuli, the visual cortices have reduced volume and decreased resting activation. On the other hand, individual variation in the effects of lower-level priors might be associated with structural and functional differences within the visual system.

In addition, variation in perceptual inference might be related to differences in neuromodulation that are known to be partially genetically determined. The glutamatergic (NMDA), dopaminergic and cholinergic systems can be of particular relevance here. Neuropharmacological and neuropathological studies on the psychosis spectrum suggest abnormal neuromodulation (e.g. NMDA-R hypofunction) of superficial cortical neurons, which cells are responsible for the encoding of precision of prediction errors^102^. NMDA modulation is composed of several parts and occurs on different timescales (postsynaptic excitatory potential induction, synaptic coincidence detection, synaptic input integration, plasticity and role in long-term synaptic depression or potentiation). However, neurotransmitter systems do not operate in isolation: NMDA-receptor inhibition (e.g. by ketamine) might lead to dopamine dysregulation. Relatedly, dopaminergic neurotransmission is essential for adaptive learning as it modulates the precision of prediction errors in the cortex^103^, and in the midbrain and the striatum, it is associated with goal-relevant belief updating^104^. In addition, the precision of sensory representations is enhanced by cholinergic (but not dopaminergic or noradrenergic) stimulation, which ultimately increases weighting of sensory evidence during perceptual inference that is behaviourally reflected in enhanced perceptual discrimination performance and attenuated psychotic symptoms. In contrast, cholinergic blockade reduces the precision of sensory representations and reduces tendency toward sensory evidence, which causes hallucinatory experiences and enhances conditioned hallucinations (reviewed by^105^). Finally, increased noradrenergic (but not cholinergic) neurotransmission was shown to destabilise the perception of ambiguous stimuli by increasing cortical excitability and altering cortical connectivity^106^, while increased GABAergic activity seems to stabilise bistable perception^107,108^. As our results indicate that our task reliably captures stable differences, it could be a suitable tool in future psychopharmacological studies of perceptual inference.

Finally, based on the predictive coding accounts of psychosis, one may also speculate how the observed differences in task performance would relate to psychosis-spectrum traits and symptoms. Some studies have implicated that psychotic phenomena are related to the dominance of expectations in perceptual inference (i.e. strong reliance on priors)^109–111^ while others have found that perceptual anomalies and hallucinations in patients with schizophrenia are related to increased sensitivity to sensory evidence (i.e. likelihood)^112^. We conducted exploratory analyses to investigate the association between task performance and schizotypal traits that may facilitate hypothesis generation. Here, we did not find stabil association between positive schizotypy and any task-related measure, which might be due to assessment: the *Unusual Experiences* subscale of the sO-LIFE questionnaire does not differentiate between proneness to hallucinations vs. delusions, even though they may have opposing correlates. Davies^110^ has shown that people prone to delusions vs. hallucinations differ in terms of extracting sensory evidence (on the task of deciding whether dots are inside or outside of previously seen, but noisy shapes): those who were prone to hallucinations were more able to extract sensory evidence, and this was especially true if they were not prone to delusions. In turn, those who were prone to delusions, especially those who were not prone to hallucinations, performed worse, suggesting that lower- vs. higher-level expectations are differentially associated with aspects of positive symptoms/schizotypy. Different scales are available to measure these constructs, e.g. the Cardiff Anomalous Perceptions Scale^113^ and the Peters’ Delusion Inventory^114^. Interestingly, individuals showing high levels of disorganised schizotypy were less likely to respond slower on ambiguous trials, which may indicate increased reliance on priors and reduced sensory precision. On a related note, disorganisation has been linked to a deficit of visual perceptual organisation, probably indicating impaired modulation by contextual expectations^115^. To sum up, future, high-powered studies should not only use reliable tools to measure individual variation in perceptual inference, but should also perform a comprehensive assessment of psychosis-related phenotypes.

## Conclusions and future directions

Overall, we conducted an experiment where higher- and lower-level effects (associative learning and priming) on perceptual inference were both present. We found that predictive processes can be captured by response tendencies and reaction times, furthermore, that for some auditory (but not visual) associative cues dominantly influence perceptual decisions about uncertain visual targets. Individual differences were evident in the data and the reliability of such differences were very high. We argued that the dominance of auditory cues might be due to downweighting of the visual modality due to the presence of uncertain visual targets. This explanation could be tested with a ‘reversed’ experimental design where perceptual decisions would be set to be made about ambiguous auditory targets that are preceded by auditory and visual cues. If our assumptions about uncertainty-driven downweighting a modality are correct, one would predict reversed effects, that is, stronger visual dominance.

We conducted exploratory analyses regarding association with schizotypal traits, which may open up novel research directions. Prior to moving forward to combining the paradigm with neuroimaging and administering it to individuals facing mental health problems, a next logical step would be the explicit mathematical modelling of the underlying computational processes (e.g. uncertainty-weighting). The idea that perception is the inferred posterior estimate of expectations and sensory evidence makes it possible to model the underlying calculations, based on prior and likelihood distributions. Uncertainty, precision and volatility are properties of the environment which can be characterized in a Bayesian framework. It has been shown that the Hierarchical Gaussian Filter^116^ is suitable for accessing individual parameters of associative learning in perceptual decision tasks with varying levels of uncertainty^8,31^. Now that we established that this paradigm is capable of identifying trait-like differences at the behavioural level, the next step is to see whether they can be explained by diverse computational mechanisms, which are based on detailed temporal information. Furthermore, this will also allow examining the temporal stability of computational parameters.

## Supplementary Information

Supplementary Information.
