# Supplementary material for: Cross-modal auditory priors drive the perception of bistable visual stimuli with reliable differences between individuals
Source: Sci Rep. 2021 Aug 20;11:16943. doi: 10.1038/s41598-021-96198-7 (PMC8379237; doi:10.1038/s41598-021-96198-7)
Supplement: Supplementary file 1 — Supplementary Information. [file 41598_2021_96198_MOESM1_ESM.docx]

Cross-modal auditory priors drive the perception of bistable visual stimuli with reliable differences between individuals

**Supplementary Materials**

Authors:

Zsófia Pálffy^1^*, Kinga Farkas^2^, Gábor Csukly^2^, Szabolcs Kéri^1,3^, Bertalan Polner^1^

^1^ Department of Cognitive Science, Budapest University of Technology and Economics, Budapest, Hungary

^2^ Department of Psychiatry and Psychotherapy, Semmelweis University, Budapest, Hungary

^3^ National Institute of Psychiatry and Addictions, Budapest, Hungary

***corresponding author: Zsófia Pálffy**

E-mail: zsofiapalffy@edu.bme.hu

Address: 1 Egry József utca, Building T, Floor 5, Department of Cognitive Science, Budapest University of Technology and Economics, Budapest 1111, Hungary

|  | Introvertive Anhedonia | | Cognitive Disorganisation | | Unusual Experiences | |
| --- | --- | --- | --- | --- | --- | --- |
| session | 1 | 2 | 1 | 2 | 1 | 2 |
| items | 10 | | 11 | | 12 | |
| mean | 19.2 | 18.5 | 32.0 | 30.4 | 28.0 | 25.7 |
| SD | 5.95 | 5.32 | 7.76 | 7.57 | 6.11 | 5.72 |
| min | 11 | 11 | 15 | 14 | 15 | 12 |
| max | 39 | 32 | 46 | 44 | 39 | 37 |
| kurtosis | 1.94 | -0.46 | -0.72 | -0.71 | -0.85 | -0.20 |
| skewness | 1.17 | 0.62 | -0.12 | -0.22 | -0.02 | -0.02 |
| Cronbach's α | 0.76 | 0.74 | 0.78 | 0.84 | 0.82 | 0.82 |
| test-retest *rho* | 0.86 | | 0.87 | | 0.92 | |
| ICC | 0.94 | | 0.95 | | 0.96 | |

**Supplementary Table 1. Descriptive statistics of the schizotypy questionnaire.**We measured positive, negative and disorganised dimensions of schizotypal traits with the Oxford–Liverpool Inventory of Feelings and Experiences, short version (sO-LIFE ^1^ , Hungarian version^2^) in both sessions. Positive schizotypy was measured with the Unusual Experiences subscale, negative schizotypy was measured with the Introvertive Anhedonia subscale, and disorganised schizotypy was assessed with the Cognitive Disorganisation subscale. Studies have shown that the sO-LIFE has appropriate psychometric properties to assess schizotypal traits in the non-clinical population^3^ (except for the Impulsive Nonconformity dimension, that we therefore did not use), which is replicated in our sample. We report sample mean, standard deviation (SD), minimum (min) and maximum (max) values, kurtosis, skewness and Cronbach’s split-half reliability (*alpha*) for every subscale in every session. Please note that instead of the original yes/no response format, in this study, to improve reliability, we administered responses using a Likert-format (1 - 5). In the last rows we report Spearman’s rank order correlation coefficients (*rho*) and intraclass correlation coefficients (ICC3,k) between sessions.

1. Mason, O., Linney, Y. & Claridge, G. Short scales for measuring schizotypy. *Schizophr. Res.* **78**, 293–296 (2005).

2. Kéri, S. Solitary minds and social capital: Latent inhibition, general intellectual functions and social network size predict creative achievements. *Psychol. Aesthet. Creat. Arts* **5**, 215–221 (2011).

3. Fonseca-Pedrero, E., Ortuño-Sierra, J., Mason, O. J. & Muñiz, J. The Oxford–Liverpool Inventory of Feelings and Experiences short version: Further validation. *Personal. Individ. Differ.* **86**, 338–343 (2015).
